# Aggravating Effects of Psychological Stress on Ligature-Induced Periodontitis via the Involvement of Local Oxidative Damage and NF-*κ*B Activation

**DOI:** 10.1155/2022/6447056

**Published:** 2022-02-16

**Authors:** Qiang Li, Yajuan Zhao, Daokun Deng, Jiuhui Yang, Yongjin Chen, Jia Liu, Min Zhang

**Affiliations:** ^1^State Key Laboratory of Military Stomatology & National Clinical Research Center for Oral Diseases & Shaanxi International Joint Research Center for Oral Diseases, Department of General Dentistry & Emergency, School of Stomatology, The Fourth Military Medical University, Xi'an, 710032, China; ^2^State Key Laboratory of Military Stomatology & National Clinical Research Center for Oral Diseases & Shaanxi Engineering Research Center for Dental Materials and Advanced Manufacture, Department of Periodontology, School of Stomatology, The Fourth Military Medical University, Xi'an, 710032, China; ^3^Department of Stomatology, 966 Hospital of PLA, Dandong 118000, China; ^4^State Key Laboratory of Military Stomatology & National Clinical Research Center for Oral Diseases & Shaanxi Clinical Research Center for Oral Diseases, Department of Orthodontics, School of Stomatology, The Fourth Military Medical University, Xi'an, 710032, China

## Abstract

Periodontitis is the leading cause of tooth loss in adults, and psychological factors play an important role in the development of periodontitis. To elucidate the adverse effects of psychological stress on the inflammatory process and redox status of periodontitis tissue, fifty male Sprague-Dawley rats were divided into the control, experimental periodontitis, psychological stress, experimental periodontitis plus psychological stress, and experimental periodontitis plus psychological stress plus fluoxetine groups. Chronic unpredictable mild stress (CUMS) was used to establish psychological stress, and silk ligature was used to induce experimental periodontitis. Four weeks later, stressed rats showed altered behaviour, serum hormone levels, and sucrose preference. More obvious alveolar bone loss and attachment loss and higher protein expressions of inflammatory cytokines were observed in the experimental periodontitis plus psychological stress group. The combination of CUMS and periodontitis had synergistic effects on increasing hypoxia-inducible factor-1*α* (HIF-1*α*) protein expression and reactive oxygen species (ROS) and malondialdehyde (MDA) contents and decreasing antioxidant enzyme activities compared with those in the stress or periodontitis groups. Moreover, psychological stress further increased p-I*κ*B*α* and p-NF-*κ*B p65 protein levels and decreased I*κ*B*α* protein levels in periodontitis rats. Fluoxetine administration alleviated the adverse effects of psychological stress on the progression of periodontitis in rats. These results hint us that psychological stress could aggravate inflammation in periodontitis tissues, which may be partly due to local worsening of oxidative damage and further activation of the nuclear factor kappa-B (NF-*κ*B) signalling pathway.

## 1. Introduction

As a chronic inflammatory disease, periodontitis is the main cause of tooth exfoliation due to severe inflammatory reactions and periodontal tissue destruction [1]. Although it is currently widely accepted that bacteria are the main aetiologic factor in periodontal disease [2], it cannot be ignored that the local, systemic, and behavioural host conditions that affect the resistance of the host to infecting periodontal microorganisms could modify the onset and progression of periodontitis [3, 4].

Psychologic condition, especially psychological stress, plays an important role in daily life and can be caused by a variety of stressors that potentially threaten an individual's homeostasis, well-being, overall health, or survival [5]. The association between psychological stress and periodontitis has been largely demonstrated recently [6, 7, 8] and has been identified as a potential risk factor for periodontal disease in some observational studies [9, 10]. Also, researchers established a conditioned fear stress model and physical restraint model in rats to induce psychological stress and confirmed that stress is associated with the progression of alveolar bone loss with altered RANK and RANKL expressions as well as quality of alveolar bone [11, 12]. Some studies even indicated that psychosocial stress has a greater influence than pathogenic bacteria on the severity of periodontal inflammation [13]. We previously observed the phenomenon of a delayed periodontitis healing process in psychologically stressed rats induced by chronic unpredictable mild stress (CUMS) [14]. CUMS procedure has been widely used in rodents and could well mimic the human experience of various stresses in daily life [15, 16]. Therefore, elucidating its important role in the progression of periodontitis will further enrich our knowledge of the pathological process of periodontal disease.

Long-term stress can decrease blood oxygen saturation and increase oxygen consumption, thereby exposing tissues to hypoxia [17]. Microcirculation impairments involving congestion of the venous bed and local bleeding into the periodontal tissue caused by psychological stress also suggest that the periodontium may undergo a shift from normoxia to hypoxia when inflammation is initiated and progresses [18]. Redox homeostasis is disturbed under anaerobic conditions, resulting in an imbalance between the oxidant and antioxidant defence systems. This leads to oxidative stress, which has been demonstrated to damage periodontal health [12, 19, 20]. Nuclear factor kappa B (NF-*κ*B) is a transcription factor with numerous biological functions, including regulating the expression of various proinflammatory factors involved in inflammation [21]. Among the variety of NF-*κ*B agonists, free radicals play an important role in contributing to the activation of NF-*κ*B [22], which is associated with hyperinflammatory responses and inflammation-induced injury in periodontitis [23]. However, the local redox state and NF-*κ*B expression in periodontitis rats exposed to psychological stress remain unclear, which might limit our understanding of the mechanism underlying the psychological aetiology of periodontitis.

Therefore, in the present study, we induced the CUMS model to investigate the effects of psychological stress on the existing periodontal damage. In addition, the local redox state and NF-*κ*B expression in the periodontium as well as their potential roles in stress conditions were explored. Furthermore, the antagonistic effect of fluoxetine on alleviating the aggravation of periodontal destruction induced by psychological stress was verified.

## 2. Materials and Methods

### 2.1. Animals and Grouping

**Figure 1:**
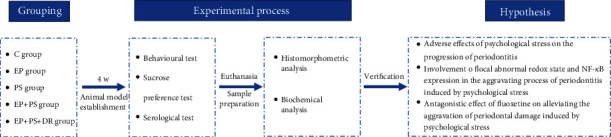
Experimental scheme. C: control rats; EP: rats with experimental periodontitis; PS: rats exposed to psychological stress; EP+PS: rats with experimental periodontitis exposed to psychological stress; EP+PS+DR: rats with experimental periodontitis exposed to psychological stress and treated with fluoxetine.

Fifty male Sprague-Dawley rats (8 weeks old, 210-230 g) were obtained from our university. They were randomly divided into five groups of 10 rats each, i.e., the C group, which comprised control rats; the EP group, which comprised rats with experimental periodontitis; the PS group, which comprised rats exposed to psychological stress; the EP+PS group, which comprised rats with experimental periodontitis exposed to psychological stress; and the EP+PS+DR group, which comprised rats with experimental periodontitis exposed to psychological stress and treated with fluoxetine. All rats were housed in a temperature-controlled room at 22 ± 1°C with a 12-hour light/dark cycle (lights on from 08:00 to 20:00) and food and water available ad libitum. Before the beginning of the experiment, the animals were acclimated to the laboratory conditions for 1 week. This study was performed in strict accordance with the recommendations of the National Institutes of Health *Guide for the Care and Use of Laboratory Animals* (NIH Publications No. 8023, revised 1978). The protocol was approved by the Animal Research Ethics Committee of the School of Stomatology of our university (No. 2015048). Every effort was made to prevent animal suffering at each stage of the experiment. The experimental scheme is shown in Figure 1.

### 2.2. Establishment of Animal Models and Drug Administration

The rats in the EP group, the EP+PS group, and the EP+PS+DR group were anaesthetized by intraperitoneal injection of 1% sodium pentobarbital (3.5 mg/kg) prepared in sterile saline, and a 4-0 silk ligature was placed around the cervix of the right second maxillary molar to induce experimental periodontitis as previously described [14]. The ligature retained oral microorganisms and remained fixed until the end of the experiment. The CUMS protocol was adapted from the procedure described by Cui et al. [24]. The rats in the PS group, the EP+PS group, and the EP+PS+DR group were subjected to seven different stressors, i.e., food deprivation (12 h), water deprivation (12 h), damp sawdust (24 h), restraint stress (1 h), immersion in cold (4°C) water (5 min), immersion in hot (45°C) water (5 min), and inversion of the light/dark cycle. Each day, a random stressor was applied, and the same stressor was used only once per week to avoid stress habituation in the rats. Fluoxetine (5 mg/kg, Eli Lilly, Indianapolis, IN, USA) was dissolved in saline and administered to the rats in the EP+PS+DR group by gavage every day. The dose of fluoxetine was determined based on a report by Bonilla-Jaime et al. [25]. The experiment lasted for 4 weeks, and the behavioural test, the sucrose preference test, serology, morphological observation, and biochemical detection were performed for all the animals in each group at the end of the experiment.

### 2.3. Open Field Test

An open field chamber (RD1412-OF, Shanghai Mobile Datum Information Technology Co., Shanghai, China) consisting of a 100 cm × 100 cm × 80 cm Plexiglas box was used in this test. This chamber was placed in a temperature-controlled room and was illuminated by one fluorescent light suspended over the chamber. Each rat was monitored for 15 min by a digital video camera to quantify the time spent in the centre and the total distance travelled. After each test, the maze was cleaned with 20% alcohol to eliminate the odour and other traces of the previously tested rat [24].

### 2.4. Sucrose Preference Test

After the open field test, the sucrose preference test was performed as previously described [26] at the end of this study. Prior to the test, the animals were placed in individual cages and deprived of food and water for 24 h. Then, each rat received 1 bottle of water and 1 bottle of 1% sucrose. The volumes of the sucrose solution and water consumed were recorded over a 24 h period. Sucrose preference, which indicates anhedonia, was defined as the ratio of the volume of sucrose consumed to the total volume of sucrose and water consumed.

### 2.5. Sample Preparation

Twenty-four hours after the sucrose preference test, the rats were anaesthetized by intraperitoneal injection of sodium pentobarbital (50 mg/kg body weight). Blood samples were obtained from the retinal vein immediately for centrifugation (4°C, 4000 rpm, 15 min) when the rats were completely anaesthetized and then stored at -80°C until serum assays were performed. After blood sample collection, the rats were sacrificed by cervical dislocation. The gingival tissue around the right second maxillary molar was immediately removed carefully and preserved at -80°C for further biochemical analysis and Western blotting. The right maxillae were immediately removed and fixed in 10% formalin for >48 hours. Subsequently, decalcification was carried out in 15% EDTA solution at room temperature for 6 weeks. Five-micron-thick paraffinized tissue sections obtained along the mesial-distal axis were stained with haematoxylin and eosin (H&E) for histomorphometric analysis.

### 2.6. Serological Assay

Aspirated serum aliquots were used to measure corticosterone (CORT) and adrenocorticotropic hormone (ACTH) contents using rat ELISA kits (Shanghai Westang Bio-Tech Co., Ltd., Shanghai, China) according to the manufacturer's instructions.

### 2.7. Histomorphometric Analysis

Histomorphometric analysis was performed under a light microscope at 100x magnification. Alveolar bone loss and attachment loss were evaluated by measuring the distance between the cement-enamel junction and the alveolar bone crest and the distance between the cement-enamel junction and junctional epithelial attachment, respectively [8, 14].

### 2.8. Determination of Oxidative Stress Marker Levels

The levels of reactive oxygen species (ROS) (specifically H_2_O_2_) and malondialdehyde (MDA) and the activities of antioxidants (superoxide dismutase (SOD), catalase (CAT), and glutathione peroxidase (GSH-Px)) in gingival tissues were detected by corresponding assay kits (Nanjing Jiancheng Bioengineering Institute, Nanjing, China) according to the manufacturer's instructions. The optical density (OD) values were read with a microplate reader (BioTek, Winooski, VT, USA).

### 2.9. Western Blotting

Gingival tissues were lysed using NP40 lysis buffer (Elpis Biotech, Seo-gu, Daejeon, Republic of Korea) containing protease inhibitor cocktail (Sigma-Aldrich, USA). The protein concentration of the lysate was assessed by the Bradford assay (Bio-Rad, California, USA). Equal amounts of protein (20 *μ*g) were subjected to 10% sodium dodecyl sulfate-polyacrylamide gel electrophoresis (SDS-PAGE) and transferred to polyvinylidene difluoride membranes (Bio-Rad, Hercules, CA), which were incubated with primary antibodies against IL-1*β*, IL-6, TNF-*α*, IFN-*γ*, HIF-1*α*, phosphorylated I*κ*B*α* (p-I*κ*B*α*), I*κ*B*α*, phosphorylated NF-*κ*B p65 (p-NF-*κ*B p65) (1 : 1000, Santa Cruz Bio. Inc., USA), and *β*-actin (1 : 1000, Cell Signaling Technology, Beverly, MA, USA) overnight at 4°C. The membranes were then washed in Tris-buffered saline containing 0.1% Tween 20 and treated with species-specific horseradish peroxidase-conjugated secondary antibodies (1 : 5000, Santa Cruz Bio. Inc., USA) for 1 h at room temperature. The membranes were washed once more and visualized using an enhanced chemiluminescence kit (Millipore, USA). Densitometric analysis of the protein bands was performed using Bio-Rad Quantity One software (Bio-Rad, USA). All samples were run in duplicate on separate gels, and protein levels were expressed relative to that of *β*-actin in arbitrary units.

### 2.10. Statistical Analysis

All the data were analysed with SPSS 17.0 software (SPSS Inc., Chicago, IL, USA). The normal distributions of the data were tested by Q–Q plots. When uniform variance was found by Bartlett's test, significant differences between each measure were determined by one-way analysis of variance (ANOVA) and Bonferroni post hoc test. The data are expressed as the mean ± standard deviation (SD). Statistical significance was set at *P* < 0.05.

## 3. Results

### 3.1. Behavioural Alterations

**Figure 2:**
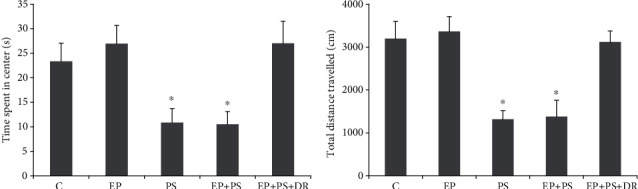
Open field test: (a) time spent in centre; (b) total distance travelled. C: control rats; EP: rats with experimental periodontitis; PS: rats exposed to psychological stress; EP+PS: rats with experimental periodontitis exposed to psychological stress; EP+PS+DR: rats with experimental periodontitis exposed to psychological stress and treated with fluoxetine. The data are expressed as *χ*^2^ ± SD, *n* = 10. ^∗^*P* < 0.05, vs. the C group, EP group, and EP+PS+DR group.

As shown in Figure 2, the PS rats and EP+PS rats spent a significantly shorter time in the centre (*P* < 0.05, Figure 2(a)) and travelled a significantly shorter distance than the control rats and experimental periodontitis rats (*P* < 0.05, Figures 2(a) and 2(b)). However, fluoxetine effectively reversed this behavioural change (*P* > 0.05).

### 3.2. Sucrose Preference Alterations

**Figure 3:**
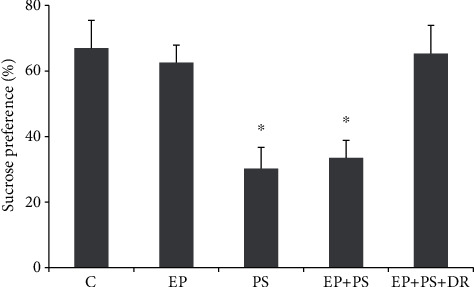
Sucrose preference. C: control rats; EP: rats with experimental periodontitis; PS: rats exposed to psychological stress; EP+PS: rats with experimental periodontitis exposed to psychological stress; EP+PS+DR: rats with experimental periodontitis exposed to psychological stress and treated with fluoxetine. The data are expressed as *χ*^2^ ± SD, *n* = 10. ^∗^*P* < 0.05, vs. the C group, EP group, and EP+PS+DR group.

As shown in Figure 3, the sucrose preference of the rats in the PS group and the EP+PS group was lower than that of the rats in the C group and the EP group (*P* < 0.05), and this change was reversed after fluoxetine administration (*P* > 0.05).

### 3.3. Serum CORT and ACTH Levels

**Figure 4:**
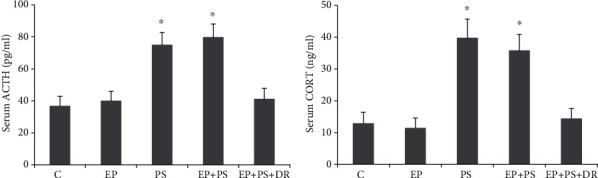
Serum CORT and ACTH assays: (a) CORT level; (b) ACTH level. C: control rats; EP: rats with experimental periodontitis; PS: rats exposed to psychological stress; EP+PS: rats with experimental periodontitis exposed to psychological stress; EP+PS+DR: rats with experimental periodontitis exposed to psychological stress and treated with fluoxetine. The data are expressed as *χ*^2^ ± SD, *n* = 10. ^∗^*P* < 0.05, vs. the C group, EP group, and EP+PS+DR group.

ELISA showed that psychological stress significantly increased serum CORT and ACTH levels in the rats in the PS group and the EP+PS group compared with those of the C group and the EP group (*P* < 0.05, Figure 4). These elevated levels of stress-related hormones returned to normal after the stress was counteracted (*P* > 0.05, Figure 4).

### 3.4. Alveolar Bone Loss and Attachment Loss Measurement

**Figure 5:**
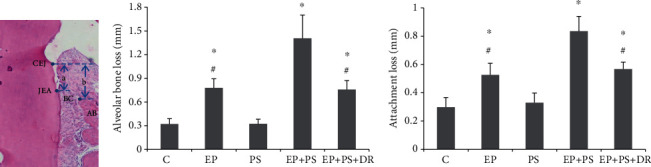
Measurement of alveolar bone loss and attachment loss: (a) schematic diagram of periodontal histological measurement; (b) alveolar resorption; (c) attachment loss. CEJ: cement-enamel junction; JEA: junctional epithelial attachment; AB: alveolar bone; BC: alveolar bone crest; a: attachment loss; b: alveolar bone loss; C: control rats; EP: rats with experimental periodontitis; PS: rats exposed to psychological stress; EP+PS: rats with experimental periodontitis exposed to psychological stress; EP+PS+DR: rats with experimental periodontitis exposed to psychological stress and treated with fluoxetine. The data are expressed as *χ*^2^ ± SD, *n* = 10. ^∗^*P* < 0.05, vs. the C group and PS group; ^#^*P* < 0.05, vs. the EP+PS group.

As shown in Figure 5, alveolar bone loss and attachment loss were increased in the periodontitis rats compared with the control rats (*P* < 0.05), and these phenomena were further exacerbated by psychological stress in the EP+PS group rats compared with the EP group rats (*P* < 0.05). However, there was no change in alveolar bone loss or attachment loss in the rats exposed to psychological stress only (*P* > 0.05). Fluoxetine eliminated the effects of stress on histomorphometry (*P* > 0.05).

### 3.5. Inflammatory Cytokine Protein Expression

**Figure 6:**
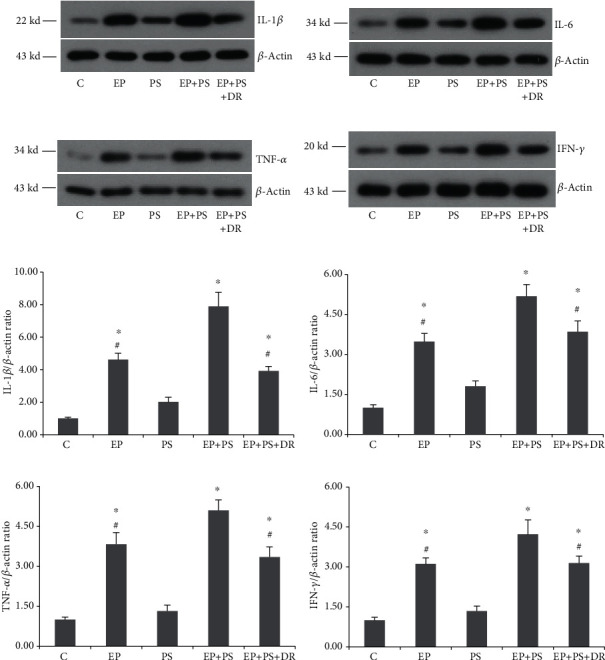
Protein expression of inflammatory cytokines: (a) protein bands of IL-1*β*; (b) protein bands of IL-6; (c) protein bands of TNF-*α*; (d) protein bands of IFN-*γ*; (e) relative protein expression of IL-1*β*; (f) relative protein expression of IL-6; (g) relative protein expression of TNF-*α*; (h) relative protein expression of IFN-*γ*. C: control rats; EP: rats with experimental periodontitis; PS: rats exposed to psychological stress; EP+PS: rats with experimental periodontitis exposed to psychological stress; EP+PS+DR: rats with experimental periodontitis exposed to psychological stress and treated with fluoxetine. The data are expressed as *χ*^2^ ± SD, *n* = 10. ^∗^*P* < 0.05, vs. the C group and PS group; ^#^*P* < 0.05, vs. the EP+PS group.

As shown in Figure 6, no obvious changes in the protein expression of IL-1*β*, IL-6, TNF-*α*, or IFN-*γ* were observed in the rats exposed to psychological stress alone compared with the rats in the C group (*P* > 0.05), whereas the protein levels of these inflammatory cytokines in periodontal tissue were increased significantly in the rats in the EP group compared to the rats in the C group (*P* < 0.05). The increases were more obvious in the rats subjected to experimental periodontitis and psychological stress simultaneously compared to the other experimental rats (*P* < 0.05). Alleviating psychological stress reversed the increase in cytokine protein expression compared to that in the rats in the EP+PS group, although there were still differences between the rats in the C group and those in the EP+PS+DR group (*P* < 0.05).

### 3.6. HIF-1*α* Protein Expression

**Figure 7:**
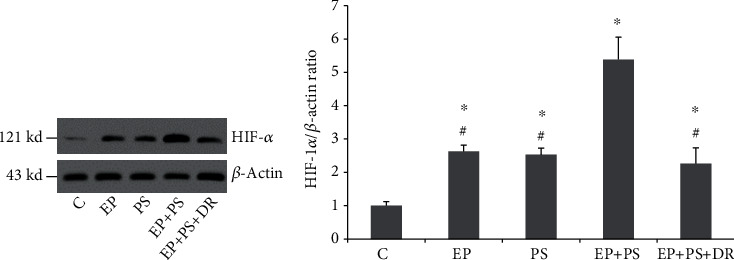
HIF-1*α* protein expression: (a) protein bands of HIF-1*α*; (b) relative protein expression of HIF-1*α*. C: control rats; EP: rats with experimental periodontitis; PS: rats exposed to psychological stress; EP+PS: rats with experimental periodontitis exposed to psychological stress; EP+PS+DR: rats with experimental periodontitis exposed to psychological stress and treated with fluoxetine. The data are expressed as *χ*^2^ ± SD, *n* = 10. ^∗^*P* < 0.05, vs. the C group and PS group; ^#^*P* < 0.05, vs. the EP+PS group.

Figure 7(a) shows the HIF-1*α* protein bands. We found that periodontitis increased HIF-1*α* protein expression in gingival tissue (*P* < 0.05, Figure 7(b)), and a similar result was also observed in the PS group rats (*P* < 0.05, Figure 7(b)). The combination of the two stimuli had a synergistic effect on increasing HIF-1*α* protein expression compared with that in the EP group and the PS group (*P* < 0.05). The differences among the EP group, the PS group, and the EP+PS+DR group were abolished when psychological stress was removed (*P* > 0.05, Figure 7(b)).

### 3.7. Oxidative Stress Indices

**Figure 8:**
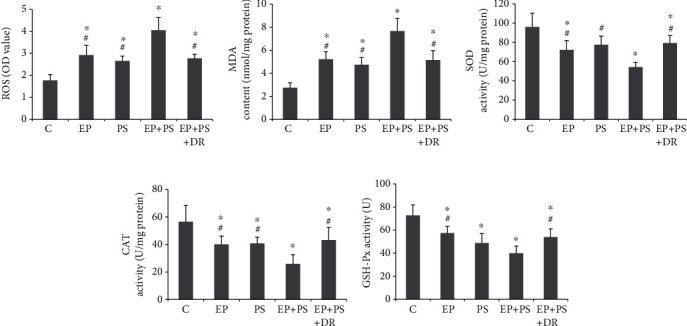
Detection of oxidative stress indices: (a) ROS content; (b) malondialdehyde content; (c) superoxide dismutase activity; (d) catalase activity; (e) glutathione peroxidase activity. C: control rats; EP: rats with experimental periodontitis; PS: rats exposed to psychological stress; EP+PS: rats with experimental periodontitis exposed to psychological stress; EP+PS+DR: rats with experimental periodontitis exposed to psychological stress and treated with fluoxetine. The data are expressed as *χ*^2^ ± SD, *n* = 10. ^∗^*P* < 0.05, vs. the C group; ^#^*P* < 0.05, vs. the EP+PS group.

As shown in Figure 8, compared with that in the control rats, increases in ROS and MDA generation were observed in the periodontitis rats and stressed rats (*P* < 0.05, Figures 8(a) and 8(b)). Psychological stress further increased the above two indices in periodontitis tissue (*P* < 0.05, Figures 8(a) and 8(b)). Meanwhile, SOD, CAT, and GSH-Px activities were decreased in both the EP group and the PS group and were decreased more obviously in the EP+PS group than in the EP group or the PS group (*P* < 0.05, Figures 8(d)–8(f)). Fluoxetine was found to play a therapeutic role in correcting the abnormal gingival redox state by reversing the increases in ROS and MDA contents and decreasing antioxidant enzyme activities (*P* < 0.05, Figure 8).

### 3.8. Expression of NF-*κ*B Signalling-Related Proteins

**Figure 9:**
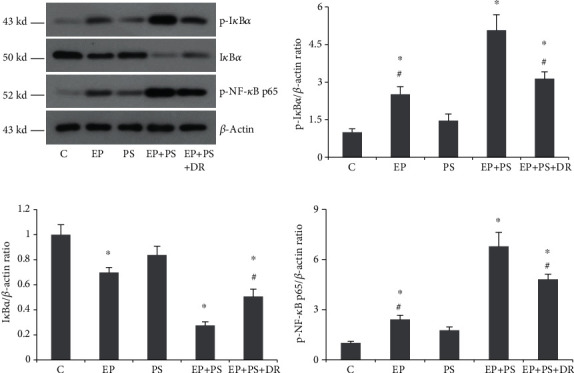
Protein expression of NF-*κ*B signalling-related proteins: (a) protein bands of p-I*κ*B*α*, I*κ*B*α*, and p-NF-*κ*B p65; (b) relative expression of p-I*κ*B*α*; (c) relative expression of I*κ*B*α*; (d) relative expression of p-NF-*κ*B p65. C: control rats; EP: rats with experimental periodontitis; PS: rats exposed to psychological stress; EP+PS: rats with experimental periodontitis exposed to psychological stress; EP+PS+DR: rats with experimental periodontitis exposed to psychological stress and treated with fluoxetine. The data are expressed as *χ*^2^ ± SD, *n* = 10. ^∗^*P* < 0.05, vs. the C group and PS group; ^#^*P* < 0.05, vs. the EP+PS group.

As shown in Figure 9, no significant differences were found in terms of the protein expression of p-I*κ*B*α*, I*κ*B*α*, or p-NF-*κ*B p65 between the control rats and stressed rats (*P* > 0.05, Figures 9(b)–9(d)). However, there were increases in p-I*κ*B*α* and p-NF-*κ*B p65 protein levels and a decrease in the I*κ*B*α* protein level in the periodontitis rats compared to the control rats (*P* < 0.05, Figures 9(b)–9(d)). Changes in the above parameters were more evident between the EP+PS rats and EP rats (*P* < 0.05, Figures 9(b)–9(d)). Fluoxetine reversed the adverse effect of psychological stress on the expression of NF-*κ*B signalling-related proteins to some extent (*P* < 0.05, Figures 9(b)–9(d)).

## 4. Discussion

This study showed that psychological stress altered the hormone and behaviour of rats. More severe periodontal injury and inflammation were observed when rats with periodontitis received psychological stress. Additionally, the combination of stress and periodontitis had synergistic adverse effects on periodontal HIF-1*α* protein expression, local redox state, and NF-*κ*B activation. The above observations could be alleviated by fluoxetine.

With the rapid development of the economy and society, people inevitably suffer from psychological stress because of increasingly fierce competition and diverse social values. Understanding the important role of psychological stress in oral health has attracted much attention from researchers and clinicians in recent years, including periodontitis which is regarded as the leading cause of tooth loss in adults. Clinical studies have shown that stressed patients exhibit more plaque accumulation, higher gingival indices, and increased levels of IL-6 and cortisol in the gingival crevicular fluid (GCF) [27]. The therapeutic outcomes of periodontal treatment alone are poor due to psychological stress [9]. The loss of alveolar bone mass in periodontal inflammatory tissues can be alleviated in animals through therapeutic interventions to combat stress [11]. All these facts indicate the important role of psychological stress in the progression of periodontitis.

The CUMS procedure used in this study utilized seven stressors, each of which was applied randomly to the rats [14, 24]. This procedure is considered not only to provide a realistic simulation of the stresses of daily life but also to effectively prevent habituation to repetitive stress [15, 16]. In the current study, the decreases of total distance moved and central zone duration in the open field test indicate that the curiosity and preference for spontaneous activities of the stressed rats were decreased [28]. Accompanying the results of increased CORT and ACTH contents as well as decreased sucrose preference, we confirmed that the CUMS protocol in this study induced a depression-like state in the PS and EP+PS animals. In addition, because it causes plaque retention, the method used in this study of tying a ligature around the teeth is widely used to establish periodontal disease in animals [8, 11, 12, 14]. Although we did not find any changes in alveolar bone loss, attachment loss, or cytokine protein expression in the gingiva of stressed rats, we confirmed that multiple stressors worsened these parameters in rats with silk ligature-induced periodontitis. These results suggest that stress alone does not disrupt periodontal morphology or affect the local periodontal inflammatory state but may promote existing destruction and inflammation. The inflammatory process induced by cytokines released by monocytes and macrophages in response to bacterial products is responsible for the breakdown of the periodontium in periodontitis [29, 30]. Recent reports have also shown that stress hormones increase gram-negative bacterial growth [31]. Hence, the acceleration of periodontal destruction after psychological stress may occur via increased cytokine production subsequent to periodontal pathogen growth.

Interestingly, in the present study, we found that CUMS had a similar effect as periodontitis in terms of increasing HIF-1*α* protein expression and ROS generation in periodontal tissue. Additionally, decreased activities of SOD, GSH-Px, and CAT, which are typical antioxidant defence enzymes, and increased content of MDA, which indicates lipid oxidation [32], were observed in the rats of the EP group and the PS group. Previous animal research has demonstrated that microcirculation impairments occur in periodontal tissue as a result of psychological stress [18]. Additionally, HIF-1*α* is an indicator of anaerobic states [33], and oxidative stress is a secondary reaction in the hypoxic microenvironment [34]. The activations of HIF-1*α* and NF-*κ*B in PDL cells and periodontal diseases are displayed under the hypoxic situation accompanied with lipopolysaccharide stimulation [35]. Therefore, it is reasonable to suppose that hypoxia occurs in the periodontium [36] when rats undergo long-term stress. Lopes Castro et al. [12] have reported oxidative imbalance in a blood sample and consider that the disequilibrium of oxidation and antioxidation could potentialize or generate alveolar bone lesions in chronically stressed rats. In the current study, we first observed that the local anomalous redox trend was worse in the inflamed gingiva following psychological stress. These findings suggest that the healthy periodontium is susceptible to oxidative stress, resulting in inflammatory reactions, and that disturbance of the redox system in the periodontium likely causes the release of more ROS and MDA as well as a decline in antioxidant activities under sustained psychological stress. Physically, a delicate balance exists between oxidant formation and antioxidant defence in organisms. However, hypoxia disturbs this balance and results in oxidative stress, which has been implicated in the pathogenesis of many diseases, including periodontitis [34]. Dental plaques induced by ligature harbour a number of bacterial pathogens that stimulate host cells to release various proinflammatory cytokines that can attract polymorphonucleocytes (PMNs) to the site of infection [37]. PMNs confront this bacterial challenge by producing proteolytic enzymes and O_2_ through an oxidative burst. This antioxidant system in the human body functions to detoxify and modify ROS into less reactive species. Destruction of the antioxidant system resulting from simultaneous inflammation and psychological stress induces severe oxidative damage to periodontal tissue. Our findings were supported by a recent report showing that stress hormones promote the expression of virulence and oxidative stress genes in *Porphyromonas gingivalis* [38].

The activation of NF-*κ*B has been shown to induce the expression of many inflammatory cytokines and is involved in many inflammatory diseases [21, 23, 39]. Basal upregulation of NF-*κ*B and its target genes has been noted in diseased periodontal ligament fibroblasts (PDLFs) compared to healthy PDLFs [23], while downregulation of NF-*κ*B may decrease the levels of cytokines and the numbers of TRAP-positive multinucleated cells in ligature-induced periodontal disease rats [39]. Levels of I*κ*B*α*, p-I*κ*B*α*, and p-NF-*κ*B p65 are typically detected to determine whether the NF-*κ*B signalling pathway has been activated [40]. In the current study, increased protein expression of p-I*κ*B*α* and p-NF-*κ*B p65 and decreased protein expression of I*κ*B*α* were observed in periodontitis tissue in the EP group rats, indicating NF-*κ*B activation. Some studies have shown stress-induced modulation of NF-*κ*B activation in certain organs and cells [41, 42]. We observed similar protein overexpression and underexpression trends of p-I*κ*B*α*, p-NF-*κ*B p65, and I*κ*B*α* in the PS group rats, but the significant differences were not confirmed. However, the promoting effect of activating NF-*κ*B was found by the evidence that the above changes were more evident when comparing the EP+PS rats with EP rats. To the best of our knowledge, this is the first study to assess the effect of psychological stress on NF-*κ*B signalling in an experimental periodontitis rat model. Jia et al. [43] demonstrated that sustained activation of NF-*κ*B by ROS is involved in the pathogenesis of stress-induced gastric damage in rats. In fact, both proinflammatory factors and ROS are known to stimulate the transcription factor NF-*κ*B [44, 45]. One might therefore speculate that further activation of NF-*κ*B signalling occurs in the periodontitis gingiva of stressed rats, in which severe inflammatory reactions and oxidative damage are observed. Alveolar bone homeostasis depends on the functional balance between osteoblasts and osteoclasts [46]. With the development of periodontitis, alveolar bone exhibits bone resorption as a result of abnormal bone remodelling. Previous studies have reported that proinflammatory cytokines driven by NF-*κ*B are powerful signals that modulate osteoblast and osteoclast activities [47], and NF-*κ*B signalling activation in osteoclasts is crucial for the differentiation and activation of these cells [48].

Fluoxetine is a classical antidepressant agent. We used fluoxetine as an antagonist of CUMS-induced depression in rats and found that its administration reversed abnormal serum hormone levels, changes in sucrose preference, and behavioural alterations. Moreover, fluoxetine alleviated the aggravation of periodontal destruction induced by psychological stress, although its effects on local inflammation, oxidative damage, and NF-*κ*B overexpression were not elucidated. Our results suggest that antidepressant therapies that are able to modulate the brain-neuroendocrine-immune system could be used as adjuvants for periodontal disease management [49]. Additionally, treatments that suppress oxidative stress and activate NF-*κ*B signalling could be important agents for treating inflammation-associated periodontal disorders.

Psychological stress is involved in the HPA axis activation which exerts a consistent regulatory influence on peripheral inflammation [50, 51]. Based on the present findings of aggravating periodontal lesions and increased expression of inflammatory cytokine in stressful states, it is interesting to discuss the potential role of the HPA axis in the future study. Meanwhile, except for depression induced in this study, anxiety is another common stressful mood in daily life. Thus, further study should also determine the effects of anxiety on the pathological process of periodontitis, and this will benefit us for further understanding the role of psychology in the aetiology of periodontitis.

## 5. Conclusions

In conclusion, psychological stress alone does not cause alveolar bone loss or attachment loss but can aggravate existing periodontitis, which might be associated with the involvement of local oxidative damage and NF-*κ*B signalling activation. Therefore, to achieve better outcomes, dentists should take psychological factors into consideration when treating patients with periodontal disease in the clinic.

## Data Availability

Data is available on request from Dr. Qiang Li (lqaq726@163.com).
